# Implantable tibial neuromodulation with an Anklet for overactive bladder: a review

**DOI:** 10.1007/s00345-026-06740-3

**Published:** 2026-09-07

**Authors:** J. F. Timmermans, J. P. F. A. Heesakkers

**Affiliations:** Department of Urology, Maastricht University Medical Centre, P.O. Box 5800, Maastricht, 6202 AZ The Netherlands

**Keywords:** Overactive bladder, Urgency urinary incontinence, Implantable tibial neuromodulation; Wearable ankle stimulator

## Abstract

**Introduction:**

Overactive bladder (OAB) is a prevalent chronic condition associated with substantial impairment in quality of life (QoL). Although conservative and pharmacological therapies remain first- and second-line treatment options, long-term adherence and efficacy are often limited by variable treatment response. Implantable tibial neuromodulation (iTNM) using an external wearable ankle stimulator has emerged as a minimally invasive therapeutic strategy combining the durability of implantable neuromodulation with the convenience of wireless home-based therapy. This review provides an overview of currently investigated iTNM systems incorporating an external wearable stimulator anklet for the treatment of OAB.

**Methods:**

A narrative review of the literature was performed on iTNM systems using an external anklet stimulator. Studies evaluating clinical efficiacy, safety, durability, device characteristics and patient-centered outcomes were reviewed.

**Results:**

14 studies were included. Three iTNM systems incorporating an external external anklet stimulator were identified: the Urgent-SQ, Protect PNS, and the Revi system (formerly RENOVA iStim). Clinically meaningful improvements were observed in urgency symptoms, urinary frequency, urge urinary incontinence (UUI) episodes, and quality of life (QoL). Safety outcomes were generally favorable, with adverse events (AEs) predominantly mild and transient. Implant migration or dislocation, revision procedures, and device-related serious adverse events (SAE) were infrequently reported.

**Conclusion:**

Current evidence suggests that iTNM using an external anklet stimulator may provide an effective and minimally invasive treatment option for patients with refractory OAB and UUI, with encouraging safety and durability outcomes. Although further comparative and long-term studies are warranted, iTNM appears to be an relevant addition to the third-line OAB management.

J.P.F.A. Heesakkers: 0000-0003-1570-1945.

## Introduction

Overactive bladder (OAB) is a symptom syndrome characterized by urinary urgency, usually accompanied by daytime urinary frequency and nocturia, with or without urgency urinary incontinence (UUI) [1]. The symptoms occur in the absence of urinary tract infection (UTI) or other identifiable underlying pathology. OAB symptoms are chronic in nature and are associated with substantial impairment in quality of life, negatively affecting social functioning and emotional well-being [2, 3]. OAB affects approximately 20% of the global population, with prevalence increasing over recent decades. Higher prevalence rates have been reported in women, older individuals, and patients with overweight or obesity [4].

First-line management consists of conservative treatment strategies, including behavioral therapies, dietary and fluid restriction, bladder training, and pelvic floor muscle training [2, 5, 6]. Although these interventions may improve symptoms, their effectiveness often depends on sustained patient motivation and adherence. In patients with insufficient symptom control, 2nd line pharmacological treatment with antimuscarinics or β3-adrenoceptor agonists is recommended and has been shown to improve episodes of urinary urgency and UUI [7–9]. However, long-term treatment persistence remains limited because of variable therapeutic response, bothersome adverse effects, and concerns regarding cognitive impairment and dementia risk associated with prolonged anticholinergic exposure [2, 7, 10].

Patients with refractory OAB (rOAB) may subsequently be treated with third-line therapies, including intradetrusor botulinum toxin injections, percutaneous tibial nerve stimulation (PTNS), and sacral neuromodulation (SNM) [2, 11]. Minimally invasive treatment options such as botulinum toxin injections and PTNS have demonstrated efficacy in improving urgency, frequency, and UUI symptoms [11–13]. Although effective, both treatments are associated with limitations, including the risk of urinary retention following botulinum toxin injections and the substantial treatment burden of conventional PTNS, which requires repeated outpatient needle-based stimulation sessions and frequent hospital visits [13–15]. Although SNM is an established and effective treatment modality, it requires implantation of a pulse generator through two relatively invasive surgical procedures and often requires device reprogramming visits, lead revisions, or surgical battery replacement [16].

Implantable tibial neuromodulation (iTNM) using an external wearable ankle stimulator has emerged as a therapeutic strategy combining the minimally invasive nature of PTNS with the convenience and durability of implantable neuromodulation systems [17, 18]. iTNM enables wireless home-based tibial nerve modulation through an implant positioned adjacent to the tibial nerve and an external ankle-worn stimulator. In most cases the single procedure can be done in supine position under local anesthesia which makes it easier accessible for the elder OAB patients. This may reduce treatment burden and facilitate patient-tailored, on-demand therapy. This review provides an overview of iTNM systems using an external wearable ankle device that have been investigated for the treatment of OAB.

## Methods

### Search strategy

A literature search was conducted in three electronic databases: PubMed, Web of Science, and Embase. The final search was performed on 22 April 2026. The search strategy combined both free-text terms and Medical Subject Headings (MeSH) related to iTNM and overactive bladder symptoms. Key search terms included combinations of tibial neuromodulation, tibial nerve stimulation, neuromodulation, implantable, overactive bladder, lower urinary tract symptoms (LUTS), urinary urgency, and urgency urinary incontinence. No restrictions were applied regarding publication date. In Embase, the “no study abstract” filter was applied to exclude conference abstracts.

### Eligibility criteria

Studies were considered eligible if they met the following criteria: original research articles involving human subjects, patients diagnosed with lower urinary tract symptoms (LUTS), OAB, urinary frequency (UF), or urinary urgency, UUI and evaluation of iTNM delivered via an anklet device. Studies were included if they reported on at least one of the following outcomes: clinical efficacy, symptom improvement (OAB symptoms), feasibility, device characteristics, safety, adverse events (AEs), limitations, or predictive factors.

### Study selection and data extraction

All identified records were imported into Covidence systematic review software (Veritas Health Innovation, Melbourne, Australia), where duplicate entries were automatically and manually removed [19]. Titles and abstracts were screened for eligibility, followed by full-text assessment of potentially relevant articles. Data extraction was performed using a standardized form, including study characteristics (study design, sample size, patient population and characteristics), device characteristics (implantation technique, implant location, stimulation parameters, and technical features), and reported efficacy and safety outcomes.

## Results

### Study selection

**Fig. 1:**
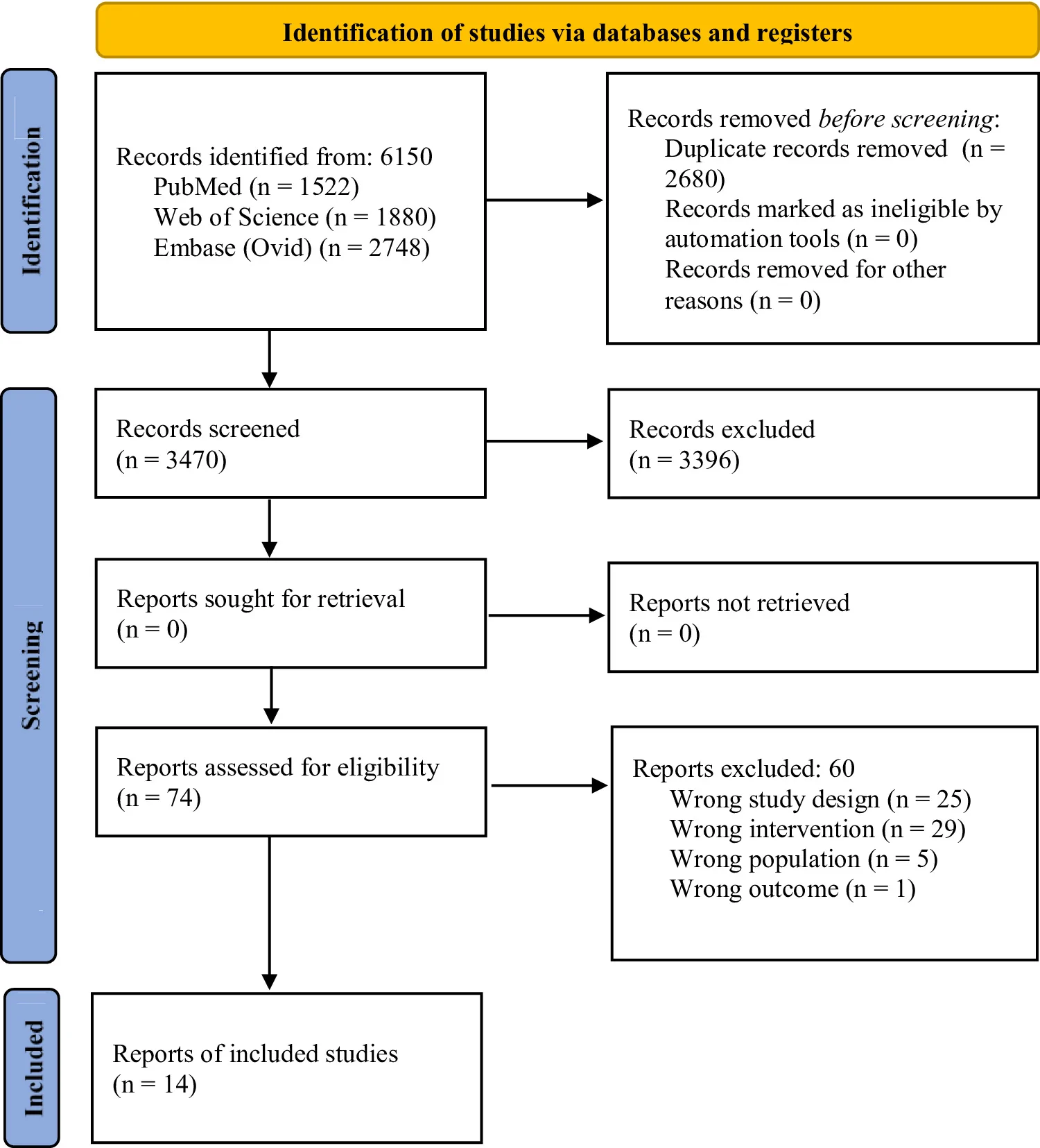
PRISMA 2020 Flow Diagram of Evidence collection in a Systematic Review on implantable tibial neuromodulation with an anklet for overactive bladder [32, 33]

The search identified a total of 6150 records. After removal of 2680 duplicates, 3470 records were screened based on title and abstract, resulting in the exclusion of 3396 records. Full-text assessment was performed for 74 articles, of which 60 studies were excluded for not meeting the inclusion criteria. Reasons for exclusion at the full-text stage included wrong study design (case reports, editorials, conference abstracts, or non-original research), irrelevant patient populations, and interventions or outcomes outside the scope of this review. Excluded studies included those evaluating non-implantable tibial nerve stimulation techniques, implantable systems without an external anklet-based stimulator, or studies primarily focused on cost-effectiveness. In total, 14 articles were included in this review [17, 18, 20–31]. An overview of the study selection process is provided in Fig. 1 using the PRISMA flow diagram [32, 33].

Three iTNM systems incorporating an external anklet stimulator were identified: the Revi system (formerly known as RENOVA iStim), Protect PNS, and Urgent-SQ. An overview of the detailed study characteristics and device specifications are presented in Tables 1 and 2.

### Urgent-SQ

**Fig. 2:**
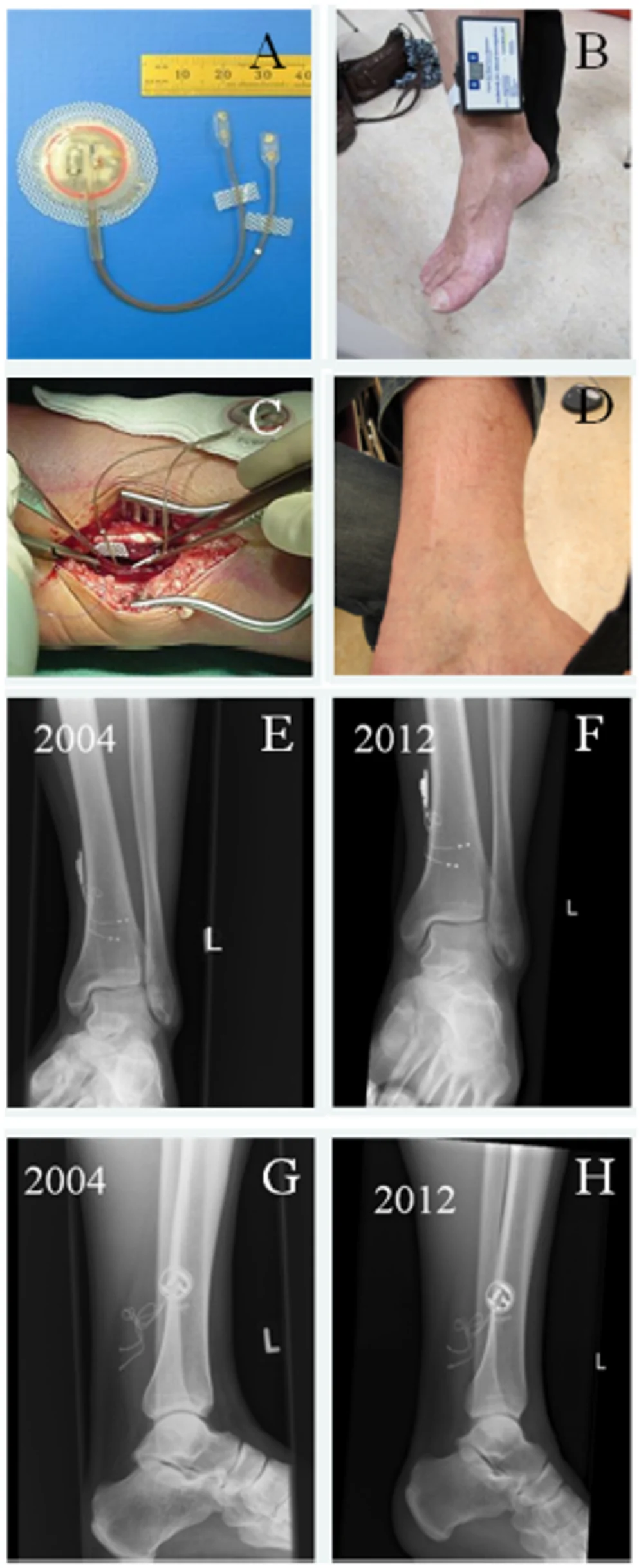
figure reprinted from Janssen et al. [24]. **A** Urgent-SQ implant consisting of a receiver plate and two electrodes positioned adjacent to the posterior tibial nerve. **B** External ankle-worn pulse generator used for on-demand stimulation. **C** Minimally invasive implantation procedure with subcutaneous placement of the receiver plate. **D** Ankle scar 9 years after implantation. **E–H** X-ray ankle, anteroposterior and lateral ankle view obtained in 2004 and 2012 demonstrating intact implants without migration, displacement or deformations after 9 year follow-up [24]

The Urgent-SQ (Uromedical) is an iTNM system that comprises an internal electromagnetic receiver connected to two monopolar electrodes and an external ankle-worn pulse generator. The internal component, measuring approximately 4 cm in diameter, is implanted adjacent to the tibial neurovascular bundle through a small incision located 5–7 cm proximal to the medial malleolus. Implantation is performed in the supine position under spinal or general anesthesia. Stimulation is delivered through radiofrequency (RF) transmission from the external pulse generator to the implanted receiver. The system is battery-free. The device and implantation procedure are presented in Fig. 2. Several studies have evaluated the efficacy and safety of the Urgent-SQ system in patients with rOAB [24, 27, 31].

The initial clinical evaluation of the Urgent-SQ device was performed by van der Pal et al. in a prospective pilot study including 8 patients with rOAB, assessing the feasibility and safety of iTNM [31]. All implantation procedures were successfully without perioperative complications. The mean operating time was 25 min. Stimulation settings included amplitudes ranging from 0 to 19 mA, pulse width of 200 µsec, and pulse rates of 12–20 Hz. Patients performed 30-minutes stimulation sessions three times weekly [31]. Significant reductions in voiding frequency and incontinence episodes, accompanied by significant improvements in I-QoL scores, were observed at 3 months and maintained at 6 months. Further reductions in incontinence episodes and improvements in SF-36 social functioning and vitality were reported at 12 months. Overall urgency-related symptoms improved based on bladder diaries. The Urgent-SQ system was generally well tolerated. Reported AEs were minor and included transient walking difficulties related to wound pain, UTIs, and spontaneous sensory sensations. No implant dislocation, erosion, local infection, revision procedures, or serious adverse events (SAEs) occurred. One patient underwent explantation because of loss of efficacy.

Janssen et al. evaluated the long-term safety, durability, and efficacy of the Urgent-SQ device in a 9-year follow-up study of the original cohort [24]. 7 out of 8 patients were available for evaluation, as one patient had previously undergone explantation. Sensory and loco-motor responses remained present in 6 of 7 patients at 9 years, and 3 of the 4 initial responders continued active therapy. Active users maintained improvements in voiding frequency and incontinence episodes, with stable I-QoL scores compared to baseline. No implant migration, structural abnormalities, infections, revisions, additional explantations, or SAEs were observed during long-term follow-up. Overall, the device was well tolerated, with minimal reported discomfort consisting of sporadic sensory sensations and occasional localized ankle discomfort.

The 18-year follow-up study by te Dorsthorst et al. demonstrated that none of the patients continued active therapy at long-term follow-up [27]. Treatment discontinuation was attributed to external stimulator failure in 40% of patients and loss of efficacy in 60%. Following discontinuation, all patients reported worsening of urgency and urge urinary incontinence symptoms. In the single patient evaluated in-hospital, no sensory or loco-motor responses could be elicited during test stimulation. No additional long-term safety concerns were identified. These findings highlight the favorable long-term safety profile of the implant, while also underscoring the importance of durable technical support and continued availability of external device components [27]. Detailed efficacy and safety outcomes are summarized in Tables 3 and 4.

Although the initial clinical findings were encouraging, the Urgent-SQ system never obtained CE certification and was therefore never introduced into routine clinical practice. Consequently, no further large-scale clinical studies or post-market data have become available.

### Protect PNS

**Fig. 3:**
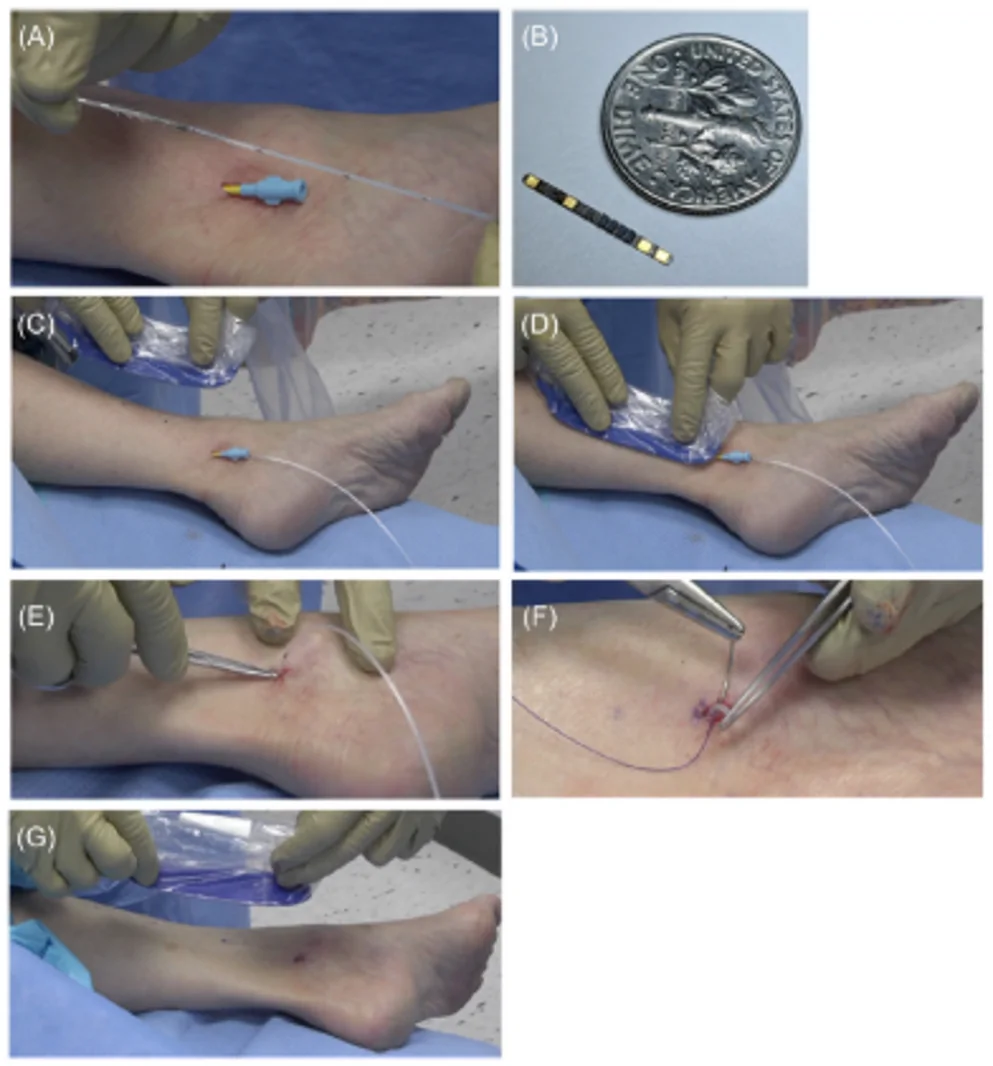
reprinted from Sirls et al. [17]. Retrograde implantation of the Protect-PNS system involves identification of anatomical landmarks above the medial malleolus, followed by subfascial introducer placement adjacent to the tibial nerve. **A** The Protect-PNS device, consisting of a miniature IPG and quadripolar electrode array, is advanced through the introducer sheath. **B** Image of miniature IPG with size comparison. **C-D** External power source used to confirm stimulation with appropriate motor/sensory response. **E-F** A distal pocket is created and the device is secured to prevent migration. **G** Wireless stimulation is confirmed again [17]

**Fig. 4:**
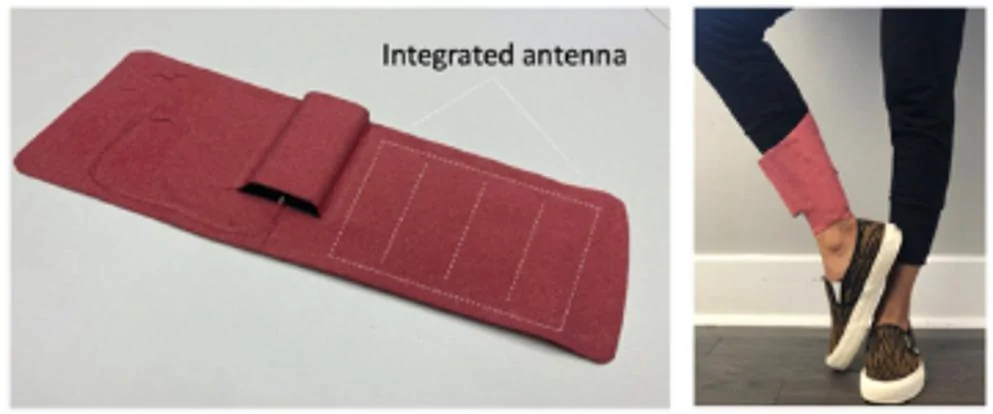
reprinted from Sirls et al. [17]. A rechargeable transmitter with the wireless antennae woven into fabric of lower leg wrap [17]

The Protect PNS system is an iTNM device consisting of a percutaneous implantable pulse generator (pIPG) with integrated quadripolar electrodes positioned parallel to the tibial neurovascular bundle. The device is implanted using a minimally invasive retrograde technique through an incision between the medial malleolus and Achilles tendon under local anesthesia in an office-based setting (Fig. 3). Stimulation is delivered via wireless energy transfer from an external rechargeable lower-leg wrap, as illustrated in Fig. 4 [17].

The randomized pilot study of Sirls et al. evaluated the safety and efficacy of the Protect PNS system in females with rOAB [17]. 9 patients were enrolled in the study, of whom 7 underwent implantation. Efficacy was assessed at 1, 4, 8 and 13 weeks, and at 6 and 12 months, while safety outcomes were evaluated up to 30–48 months. The mean procedure time was 27 min. Sensory and loco-motor responses were confirmed intraoperatively to verify appropriate device positioning. Patients performed stimulation for 6–8 h daily, although detailed stimulation parameters were not reported. Clinical improvement was observed early after implantation, with a more than 50% reduction in UUI episodes after 1 week of treatment. At 12 months, mean UUI episodes per day decreased from 3.17 at baseline to 0.83, representing a 74% reduction, while 50% of patients were completely dry. These improvements were accompanied by enhancement in patient-reported outcomes, including OAB-q and I-QoL scores. Clinical outcomes are summarized in Table 3. The overall safety profile was favorable, with no implant migration, infections, or SAEs reported. Mechanical failure of two first-generation pIPG devices required explantation and redesign of the device to a length of < 7 cm, followed by successful reimplantation. Safety outcomes are summarized in Table 4.

A prospective multicenter randomized controlled non-inferiority trial comparing Protect PNS with InterStim sacral neuromodulation in patients with refractory OAB symptoms has been registered (PROTECT trial, ClinicalTrials.gov identifier: NCT02577302) [9]. The study was initiated in 2015 and was expected to be completed in 2025. However, no clinical outcomes have been published to date.

### The Revi system

**Fig. 5:**
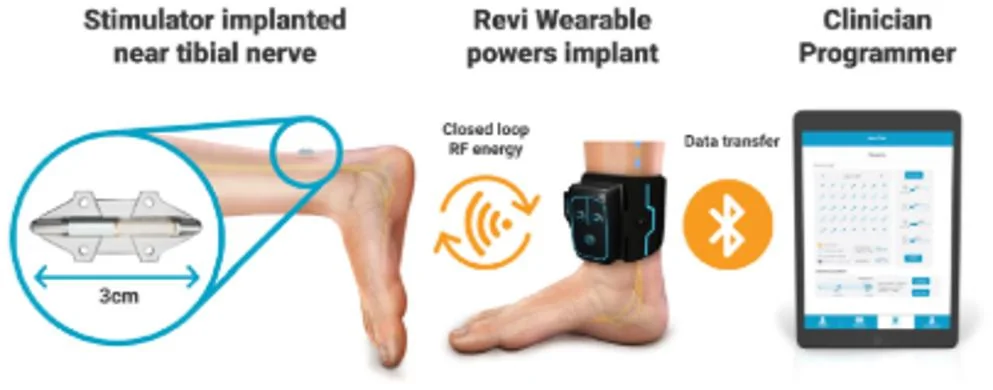
reprinted from Heesakkers et al. [18]. Device design of the Revi system. The implant is placed above the ankle through a small incision and secured to the fascia after intraoperative testing. An external ankle-worn unit delivers stimulation to the tibial nerve via wireless magnetic coupling [18]

The Revi system (BlueWind Medical) is an iTNM device consisting of a cylindrical electromagnetic receiver with bipolar electrodes and fixation wings. The implant measuring approximately 30 mm x 30 mm, is positioned adjacent to the tibial neurovascular bundle through an incision approximately 3 cm superior and 2 cm posterior to the medial malleolus. Implantation is performed under local or general anesthesia. Stimulation is delivered through wireless energy transfer from an external ankle-worn control unit. The system does not contain an implanted battery. The device design is shown in Fig. 5.

The Revi system, referred to as the RENOVA iStim system in the earlier studies, was evaluated across several prospective studies in patient with rOAB with or without UUI [21, 22, 29, 30]. Van Breda et al. reported favorable short-term safety and efficacy outcomes in the initial prospective pilot study of 15 patients with 3-month follow-up [30]. Patients received stimulation sessions of 30 min performed 6 times weekly. Median surgical time was 34 min, with appropriate sensory and loco-motor responses confirmed intraoperatively in all patients. Significant improvements were observed in severe urgency episodes (6.5 to 2.0 episodes/day), voiding frequency (11.8 to 8.1 voids/day), severe UUI episodes (2.8 to 0.3 episodes/day), and urine loss on pad testing. A ≥ 50% improvement in severe urgency and severe UUI was achieved in 93% and 71% of patients, respectively. Significant improvements were additionally observed across multiple QoL measures, including the ICIQ-FLUTS, UDI, PPBC, and bother score.

The prospective interventional study by Heesakkers et al. confirmed these findings at 6 months in a larger cohort of 36 implanted patients [22]. Patients received stimulation sessions of 30 min performed 3 times weekly. Mean surgical time was 34.8 min, and appropriate sensory and loco-motor responses were confirmed intraoperatively. Significant reductions were observed in urgency episodes (7.5 to 3.8 episodes/day), voiding frequency (12 to 9.4 voids/day), UUI episodes (6.6 to 3.9 episodes/day), and pad use (3.1 to 2.2 pads/day). Overall treatment success was achieved in 70.6% of patients, while 27.6% of patients with UUI became completely dry. Significant improvements were additionally observed across all OAB-q and HRQL domains. Long-term outcomes were subsequently evaluated by te Dorsthorst et al. in a 3-year follow-up study, followed by a post-hoc analysis investigating patient-tailored treatment behavior and predictive factors for treatment response [21, 29]. During long-term follow-up, patients increasingly adopted patient-tailored stimulation regimens based on individual symptom control. Sustained and significant improvements were observed in urgency episodes (7.48 to 4.47 episodes/day), voiding frequency (12.04 to 9.38 voids/day), UUI episodes (6.6 to 3.4 episodes/day), and pad use (3.1 to 1.9 pads/day) at 36 months. Furthermore, 73% of patients demonstrated clinically meaningful HRQL improvement. In a subsequent post-hoc analysis, te Dorsthorst et al. evaluated patient-tailored treatment behavior and predictive factors for response in 32 implanted patients [29]. Considerable variability in stimulation regimens was observed, ranging from once every 6 weeks to 14 sessions per week. All patients adjusted stimulation intensity during follow-up, with non-responders requiring more frequent adjustments than responders. Responders demonstrated significantly higher stimulation amplitudes at 3 months compared to activation settings (6.7 vs. 5.8 mA, *p* = 0.049). Body mass index (BMI) was identified as the only significant negative predictor of treatment response, whereas age, sex, and previous PTNS treatment were not associated with clinical outcomes.

Overall, sustained efficacy and a favorable long-term safety profile were observed. Reported AEs were predominantly procedure-related and included prolonged antibiotic use, prolonged pain medication use, implant-site pain, wound complications, nausea, and suspected infections with negative cultures when obtained [22, 30]. One explantation due to implant-site pain and swelling was reported by Heesakkers et al. [22]. No implant migration, dislocation, device failures, revisions, or device-related SAEs were reported during long-term follow-up [21]. Detailed efficacy and safety outcomes are summarized in Tables 3 and 4.

Following the early feasibility and long-term follow-up studies performed with the RENOVA iStim system, the device was subsequently renamed the Revi system (BlueWind Medical). Thereafter, the system was evaluated in prospective studies, originating from the OASIS trial program in female patients with UUI [18, 20, 23, 28].

Initial 12-month efficacy and safety outcomes were reported by Heesakkers et al. in a prospective open-label pivotal trial including 151 implanted patients [18]. Significant improvements were observed in urgency symptoms, with PPIUS response rates of 74.0% at 6 months and 80.1% at 12 months. Furthermore, ≥ 50% reduction in UUI episodes was achieved in 76.4% and 78.4% of patients at 6 and 12 months, respectively, while 50% of patients achieved complete dryness at 12 months. Significant improvements in OAB-q scores were additionally observed, with 93.5% of patients reporting subjective symptom improvement. Long-term follow-up of the OASIS cohort was subsequently reported by Heesakkers et al. at 24 months in 121 patients, of whom 79 completed 24-month follow-up [23]. Sustained improvements were observed in urgency symptoms and voiding frequency, while UUI episodes were significantly reduced (4.5 to 1.3 episodes/day) at 24 months. 28% of patients remained completely dry, while 79% achieved ≥ 50% reduction in UUI episodes and 56% achieved ≥ 75% reduction. Significant improvements in OAB-q scores, PGI-I outcomes, and treatment satisfaction were maintained throughout follow-up. Reported AEs were predominantly mild, including stimulation-related pain, wound complications, cellulitis, numbness, and wound infections. One explantation before 9-month follow-up and 3 explanations during 24-month follow-up were reported, none considered device- or procedure-related. Detailed safety outcomes are summarized in Table 4.

Secondary analyses evaluated predictive factors for treatment response and long-term QoL outcomes [25, 28]. Kendall et al. identified larger leg circumference (≥ 23 cm), BMI ≥ 30 kg/m², and higher baseline UUI severity as negative predictors of treatment success, whereas recent OAB medication use was associated with improved clinical response [25]. Sutherland et al. demonstrated improvements in OAB-q symptom severity (70.3 to 31.0) and HRQL scores (46.6 to 83.3) at 24 months, with 87.2% of patients reporting clinically meaningful QoL improvement (28). Furthermore, PGI-I improvement, treatment satisfaction, and willingness to continue therapy were reported in 96.9%, 96.7%, and 100% of patients, respectively.

Extended 3-year efficacy and safety outcomes were recently reported by Amundsen et al. [20]. Sustained efficacy was maintained through 36 months, with ≥ 50% reduction in moderate-to-severe urgency episodes achieved in 71.1% of patients. Improvements were maintained in voiding frequency (10.0 to 8.1 voids/day), while UUI episodes were significantly reduced (4.8 to 3.6 episodes/day). Overall responder rates remained stable at 79%, with 63% of patients achieving ≥ 75% reduction in UUI episodes. Furthermore, durable improvements in OAB-q scores and patient satisfaction were maintained throughout follow-up, with 95% of patients reporting treatment satisfaction and 82% reporting themselves as “much better” or “very much better”. During 36-month follow-up, Amundsen et al. reported pain or burning sensations in seven patients, granuloma formation in one patient, and infection in one patient between 24 and 36 months. In total, five explantations were reported from baseline to 36 months, without device-related SAEs.

In addition, Kendall et al. described revision surgery outcomes in a separate retrospective case series of previously implanted OAB patients [26]. Improvements were observed in urgency episodes (5.67 to 3.53 episodes/day), voiding frequency (11.1 to 8.4 voids/day), and UUI episodes (7.7 to 3.1 episodes/day) following revision surgery, while all patients reported satisfaction with treatment outcomes. Reported safety outcomes were transient ankle edema, mild scar redness, and loss of efficacy requiring reprogramming in one patient. No SAEs were observed [26].

**Table 1:** study characteristics

First author, year	Design	Population	*N*, (% male)	Mean age, yr	Follow-up	Objectives	Key assessments
Urgent-SQ							
Van der Pal et al., 2006 [31]	Prospective pilot study	rOAB	8 (25)	56	3–12 mo	Feasibility, clinical response, safety	Voiding diary, QoL, safety
Janssen et al., 2013 [24]	Open-label long-term FU	Previously implanted rOAB	7 (28.6)	65	9 year	Long-term safety, durability	Voiding diary, QoL, implant integrity
Dorsthorst et al., 2022 [27]	Observational long-term FU	Previously implanted rOAB	5 (40)	72	18 yr	Long-term safety, usability	Telephone interview, device usability
Protect PNS							
Sirls et al., 2023 [17]	Randomized pilot study	Female rOAB/ UUI	9, 7 implanted (0)	70.6	0–12 mo efficacy, 30–48 mo safety	Safety, UUI reduction	Voiding diary, QoL, safety, X-ray migration assessment
The Revi system							
Van Breda et al., 2017 [30]	Prospective pilot study	rOAB ± UUI	15 (13.3)	54	1–3 mo	Safety and clinical response	Voiding diary, pad test, QoL, safety
Heesakkers et al., 2018 [22]	Prospective interventional study	rOAB ± UUI	36 (13.9)	54.1	1–6 mo	Safety and clinical response	Voiding diary, pad test, QoL, safety
Dorsthorst et al., 2020 [21]	Long-term FU study	Previously implanted rOAB	20 (20)	56.1	18–36 mo	Long-term safety and efficacy	Voiding diary, QoL, safety
Dorsthorst et al., 2022 [29]	Secondary analysis	Previously implanted rOAB	32 (12)	58*	1–6 mo + extended FU	Predictors of response	Device logs, stimulation settings
Heesakkers et al., 2024 [18]	Prospective pivotal study	Female UUI	151 (0)	58.8	1–12 mo	Safety and efficacy	Bladder diary, QoL, AEs
Heesakkers et al., 2025 [23]	Long-term pivotal FU study	Female UUI	121 (0), 79 completed 24 mo FU	59	18–24 m	Efficacy and safety	Bladder diary, QoL, AEs
Kendall et al., 2025 [26]	Retrospective case series	Previously implanted OAB	4 (40)	73	3 mo	Feasibility of revision surgery	Bladder diary, Qol, AEs
Kendall et al., 2025 [25]	Secondary analysis	Female UUI	151 (0)	58.8	1–12 mo	Predictors of treatment success	Bladder diary, QoL, baseline predictors
Sutherland et al., 2026 [28]	Secondary analysis	Female UUI	151 (0)	58.8	6–24 mo	QoL improvement	QoL
Amundsen et al., 2026 [20]	Long-term pivotal FU study	Female UUI	119 (0), 90 completed 36 mo FU	58.8 (at baseline)	6–36 m	Long-term efficacy and safety	Bladder diary, QoL, AEs

*Median age is reported

FU= follow-up, OAB= overactive bladder, rOAB= refractory OAB, UUI= Urgency Urinary Incontinence, QoL = Quality of life, AE = adverse event, mo= month, yr= year

**Table 2:** Device characteristics

Device name	Implanted component	Size	Implant location	Implant procedure	External anklet	Communication	Stimulation parameters	Typical stimulation regimen	Implanted battery
Urgent-SQ	Electromagnetic pulse receiver with monopolar electrodes	~ 4 cm body; ~1 cm² electrodes	Adjacent to tibial neurovascular bundle proximal to medial malleolus	Open implantation, spinal of general anesthesia	Ankle-worn pulse generator	RF transmission	0–19 mA, 200 ms, 12–20 Hz	30-min, 3x/week	No
Protect PNS	pIPG with quadripolar electrodes	< 7 cm	Between medial malleolus and Achilles tendon adjacent to tibial neurovascular bundle	Office-based, local anesthesia	Lower-leg wireless wrap	Wireless energy transfer	N.R.	6–8 h/day	No
RENOVA iStim	Cylindrical electromagnetic power receiver with bipolar electrodes	25 × 3.4 mm	Adjacent to tibial nerve proximal to medial malleolus	Open implantation under general or local anesthesia	External ankle-worn control unit	Wireless energy transfer	0–9 mA, 50–800 µs, 5–40 Hz	30 min, 3–6×/week	No
Revi system^#^	Wireless implant with two electrodes and receiver	30 × 2.7 mm	Adjacent to tibial nerve proximal to medial malleolus	Open implantation, local anesthesia	External ankle-worn control unit	Magnetic coupling/wireless	0.2–10 mA, 190–790 µs, 2–30 Hz	Variable patient-tailored regimen (30–60 min/day to 30 min 2×/week)	No

^#^RENOVA iStim has been renamed as the Revi system

*RF* Radio-frequency, *pIPG* percutaneous implantable pulse generator

**Table 3:** Results

First author, year	Urgency	Frequency (voids/day)	UUI (episodes/day)	QoL outcomes	Responder rate
Urgent-SQ					
Van der Pal et al., 2006 [31]	Improvement symptoms	14.4→9.4* (3 mo), 10.9 (6 mo), 11.6 (12 mo)	9.3→3.9* (3 mo), 3.6* (6 mo), 1.9 (12 mo)	I-QoL score 64 →82.5* (3 mo), 86.5* (6 mo), 83.6 (12 mo)	≥ 50% improvement: 5/8 (3 mo), 6/8 (6 mo), 4/7 (12 mo)
Janssen et al., 2013 [24]	Not quantified	Improved in 2/3 active users	Reduced in 2/3 active users	Stable/improved I-QoL	3/4 initial responders continued therapy (9 year)
Dorsthorst et al., 2022 [27]	Symptoms worsened after discontinuation	N.R.	Symptoms worsened after discontinuation therapy	N.R.	No active users (18 year)
Protect PNS					
Sirls et al., 2023 [17]	OAB-q improved 75.2→39.7 (12 mo)	N.R.	3.17→0.83 (12 mo)	I-QoL improved 59.3→81.8 (12 mo)	74% responder, 50% dry (12 mo)
The Revi system					
Van Breda et al., 2017 [30]	Severe episodes/day 6.5→2.0*	11.8→8.1* (3 mo)	2.8→0.3* (3 mo)	Improvement in ICIQ-FLUTS*, bother score*, UDI*, and PPBC*	93% urgency and 71%, severe UUI response
Heesakkers et al., 2018 [22]	Episodes/day 7.5→3.8* (6 mo)	12→9.4* (6 mo)	6.6→3.9*	Improvement in all OAB-q* and HRQL* domains	≥ 50% improvement:70.6%*, UF 66.7%*, 27.6% dry (6 mo)
Dorsthorst et al., 2020 [21]	Episodes/day 7.48→4.47* (36 mo)	12.04→9.38* (36 mo)	6.6→3.4	73% improved HRQL above MID (36 mo)	Overall success 75% (36 m)
Dorsthorst et al., 2022 [29]	N.R.	N.R.	N.R.	N.R.	22 responders, 10 non-responders
Heesakkers et al., 2024 [18]	PPIUS response 74.0%* (6 mo), 80.1%* (12 mo)	10.0 ◊ 8.3 (12 mo)	4.8 ◊ 1.2 (12 mo)	Improvement OAB-q*, 93.5% (12 mo)	≥ 50% improvement *: 76.4% (6 mo) 78.4% (12 mo) 50% dry (12 mo)
Heesakkers et al., 2025 [23]	≥ 50% reduction: 62.9% (24 mo)	10.0→8.3 (24 mo)	4.5→1.3* (24 mo)	Improvement in OAB-q*, PGI-I* and treatment satisfaction	79% responders, 56% ≥75% UUI reduction, 28% dry (24 mo)
Kendall et al., 2025 [26]	Urgency episodes (score > 2/5) 5.67→3.53	11.1→8.4	7.7→3.1	Mean PGI-I score 3 (“slightly better”)	All patients satisfied after revision
Kendall et al., 2025 [25]	P.R.	P.R.	> 50% reduction: 63.0% (1 mo), 69.2% (3 mo) 77.8% (6 mo), 74.1% (9 mo), 82.0% (12 mo)	Baseline QoL associated with success	Negative predictors: leg circumference ≥ 23 cm, BMI ≥ 30 kg/m², large baseline leaks Positive predictor: recent OAB medication use
Sutherland et al., 2026 [28]	OAB-q symptom severity 70.3→31.0 (24 mo)	P.R.	P.R.	HRQL improved 46.6→83.3, PGI-I improvement: 96.9%	P.R.
Amundsen et al., 2026 [20]	≥ 50% reduction 71.1%,, OAB-q improvement (36 mo)	10.0→8.1 (36 mo)	4.8→3.6* (36 mo)	95% satisfied; 82% “much/very much better”	79% responders (36 mo), 63% ≥75% UUI reduction

*Compared to baseline and *p* < 0.05

*OR* operating room, *mo* months, *yr* years, *N.R.* not reported, *h* hour, *IQ-FLUTS* International Consultation on Incontinence Modular Questionnaire-Female Lower Urinary Tract Symptoms, *PPBC* Patient Perception of Bladder Condition, *UDI* Urogenital Distress Inventory, *OABq* overactive bladder questionnaire ,*HRQL* Health Related Quality of life questionnaire, *y* year, *m* month, *MID* minimal important difference, *PPIUS* Patient Perception of Intensity of Urgency Scale, *PGI-I* Patient Global Impression of Improvement questionnaires, *N.R.* Not Reported, *P.R.* Previously Reported

**Table 4:** Device-, treatment-, and procedure-related adverse events

First author, year	Pain/discomfort	Wound complications	Infection	Device failure/dislocation	Explantation	SAEs
Urgent-SQ						
Van der Pal et al., 2006 [31]	Spontaneous sensory sensations (*n* = 7), Temporary walking difficulties due to wound pain (*n* = 3)	None	UTI (*n* = 2)	Stimulator failure (*n* = 1)	1	None
Janssen et al., 2013 [24]	Sensory sensations (*n* = 1), ankle discomfort (*n* = 1)	N.R.	None	None	None	None
Te Dorsthorst et al., 2022 [27]	None	N.R.	None	None	None	None
Protect PNS						
Sirls et al., 2023 [17]	mild implant-site pain (*n* = 1)	Superficial suture exposure (*n* = 1)	None	mechanical failure (*n* = 2), tip exposure requiring revision (*n* = 2)	2	None
The Revi system						
Van Breda et al., 2017 [30]	Prolonged pain medication (*n* = 3)	Prolonged antibiotics (*n* = 3),	Suspected infection (cultures negative) (*n* = 1)	None	1	None
Heesakkers et al., 2018 [22]	Implant-site pain (*n* = 5), pain stimulation (*n* = 1), nausea (*n* = 1)	Wound complications (*n* = 3)	Suspected infection (*n* = 8)	None	1	1 explant for pain/swelling
Te Dorsthorst et al., 2020 [21]	None	None	None	None	None	None
Te Dorsthorst et al., 2022 [29]	N.R.	N.R.	N.R.	N.R.	N.R.	N.R.
Heesakkers et al., 2024 [18]	Pain stimulation (*n* = 6)	Wound complications (*n* = 10) Numbness/swollen foot/rash (*n* = 5)	Wound infection (*n* = 2), cellulitis (*n* = 1)	None	1	None
Heesakkers et al., 2025 [23]	None	None	Delayed superficial skin infection (*n* = 1)	None	3	None
Kendall et al., 2025 [26]	Ankle edema (*n* = 1)	Mild scar redness(*n* = 1)	None	Reprogramming (*n* = 1)	None	None
Kendall et al., 2025 [25]	N.R.	N.R.	N.R.	N.R.	N.R.	N.R.
Sutherland et al., 2026 [28]	P.R.	P.R.	P.R.	P.R.	P.R.	P.R.
Amundsen et al., 2026 [20]	Pain/burning sensations (*n* = 7) (24–36 m)	Granuloma (*n* = 1) (24–36 m)	Infection (*n* = 1) (24–36 m)	None	5 (baseline-36 m	None

*FU* follow-up, *UTI* urinary tract infection, *SAE* serious adverse event, *m* month, *N.R.* not reported, *P.R.* previously reported

## Discussion

The present review provides a comprehensive overview of published data of iTNM systems incorporating an external wearable ankle stimulator, including the Urgent-SQ, Protect PNS and Revi system, for the treatment of rOAB. Although the data are accessible for everyone, only the Revi system is available for routine clinical practice. Consistent clinically meaningful improvements and favorable response rates were observed. Overall, these findings suggest that iTNM may provide effective symptom control while maintaining a favorable safety profile in patients with rOAB. These findings represent a promising addition to the evolving neuromodulation landscape. The exact mechanism underlying tibial nerve stimulation remains incompletely understood. It is hypothesized that stimulation of the posterior tibial nerve modulates afferent signaling to the spinal cord, thereby influencing neural control of the lower urinary tract. The posterior tibial nerve originates from the spinal segments L4–S3, which are involved in the micturition reflex. In contrast to SNM, which typically targets only the S3 nerve root, tibial nerve stimulation may provide broader afferent activation of the spinal cord and brain. This broader neural modulation may contribute to improvement of OAB symptoms [17, 34].

The Urgent-SQ system demonstrated sustained efficacy, safety, and QoL improvements in active users up to 9 years after implantation. However, the absence of active therapy at 18-year follow-up, primarily due to loss of efficacy and external stimulator failure, highlights that durable treatment success depends not only on clinical efficacy, but also on reliable technical support and continued availability of external device components [24, 27, 31]. Similarly, the Protect PNS system demonstrated favorable efficacy and safety outcomes, including meaningful reductions in UUI episodes and complete continence in 50% of patients, although these findings were derived from a small pilot cohort [17]. Among the currently investigated systems, the Revi system has been evaluated most extensively and demonstrated durable symptom improvement with stable responder rates up to 36 months. Sustained benefits were observed across urgency symptoms, urinary frequency, UUI episodes, pad use, and quality of life, while a substantial proportion of patients achieved complete continence.

Overall, safety outcomes across studies were favorable. Reported AEs were predominantly mild, transient, and mainly occurred during the early postoperative period, most commonly consisting of implant-site discomfort, wound complications, suspected infections, transient sensory symptoms, and stimulation-related pain. Importantly, implant migration or dislocation, revision procedures, and device-related serious adverse events were infrequently reported across the investigated systems. Furthermore, long-term follow-up of the Urgent-SQ system additionally demonstrated absence of implant-related pain, swelling, or other late AEs, supporting the long-term safety profile of iTNM [18, 20–23, 29–31].

An important advantage of iTNM systems incorporating an anklet is the ability to provide wireless, home-based, and patient-tailored therapy. In contrast to PTNS, stimulation can be delivered on demand without repeated needle insertions or frequent outpatient visits. This is particularly relevant given the chronic nature of OAB and the logistical burden associated with repeated PTNS sessions, which has previously been identified as a contributor to treatment discontinuation [14, 24]. The home-based nature of iTNM may be especially beneficial for elderly OAB patients, who often experience mobility limitations and may face difficulties attending frequent hospital visits. Several studies included in this review demonstrated increasing adoption of individualized stimulation regimens over time, with stimulation frequency and intensity adjusted according to symptom control and patient preference [18, 29]. Sutherland et al. demonstrated significant improvements in health-related quality of life, treatment satisfaction, and willingness to continue therapy, suggesting that the minimally invasive nature of iTNM and reduced treatment burden may positively influence patient-centered outcomes [28]. Studies investigating predictors of treatment response may further contribute to individualized treatment strategies, patient counseling, and optimization of patient selection. Te Dorsthorst et al. identified higher BMI as a negative predictor of treatment success, whereas Kendall et al. additionally found that larger leg circumference (≥ 23 cm), BMI ≥ 30 kg/m² and large baseline leaks were negative predictors of treatment success [25, 29]. These findings suggest that anatomical factors may influence stimulation efficacy, potentially due to reduced energy transfer efficiency between the external stimulator and the implanted device.

Another important advantage of anklet-based iTNM systems is the minimally invasive implantation procedure. The systems were implanted through relatively small incisions under local anesthesia, with few perioperative complications and low reintervention rates. In addition, the investigated systems were battery-free, thereby eliminating the need for surgical battery replacement procedures. This may represent an important advantage over fully implantable neuromodulation systems, which typically require additional surgical intervention when the implanted battery approaches the end of its lifespan. iTNM implantation may therefore be advantageous in older or more frail patient population. However, Kendall et al. demonstrated that when revision surgery was required, revision outcomes in previously implanted OAB patients remained favorable. Improvements in urinary symptoms were observed following revision procedures, and patients continued to report satisfaction with treatment outcomes after reoperation [26]. Recent health-economic analyses suggest that iTNM may represent a cost-effective treatment strategy for rOAB and UUI [35]. Amundsen et al. demonstrated that iTNM using the Revi system was associated with improved quality-adjusted survival and lower overall healthcare costs compared with conservative therapy from a US payer perspective [35]. These findings align with a recent systematic review evaluating the cost-effectiveness of OAB treatments, which reported competitive economic outcomes for neuromodulation compared with conservative treatment [36]. Furthermore, iTNM may offer cost-effectiveness comparable to PTNS, potentially due to the reduced outpatient visit burden associated with home-based stimulation therapy burden [35].

Current treatment guidelines recommend intradetrusor botulinum toxin injections, PTNS, and SNM as established third-line treatment options for rOAB. Reported ≥ 50% responder rates range from 37.3% to 81.8% for PTNS [37]. A recent systematic review and meta-analysis comparing SNM with iTNM reported ≥ 50% overall OAB responder rates of 73.9% for SNM and 79.4% for iTNM [16]. However, the iTNM analysis included only the Revi system and eCoin. Unlike the anklet-based systems reviewed here, eCoin is a fully implantable, battery-powered device that delivers autonomous stimulation without an external wearable stimulator. Clinical studies have demonstrated sustained responder rates of approximately 65–70% at 12 months, although battery depletion necessitates surgical replacement of the implant [38]. In contrast, the present review specifically focuses on iTNM with anklet. Within the studies included in the present review, reported ≥ 50% responder rates ranged from 57 to 75% for the Urgent-SQ system, and 70.6–79% for the Revi system. Protect PNS demonstrated an overall responder rate of 74%. Although these findings suggest that anklet-based iTNM achieves comparable clinical outcomes to established third-line therapies, direct comparisons should be interpreted with caution because responder definitions, study populations, and follow-up durations differ substantially between studies. Direct comparative trials are therefore required to determine the relative efficacy, safety, and optimal position of anklet-based iTNM within the current treatment algorithm.

Despite the promising findings summarized in this review, several limitations should be acknowledged. Three iTNM systems incorporating an anklet have been described in the literature. However, the extent of the available clinical evidence differs considerably between these systems. Whereas the Revi system has been evaluated in several prospective studies with longer-term follow-up, evidence for the Urgent-SQ and Protect PNS systems remains limited. Urgent-SQ was primarily evaluated in early feasibility studies involving small patient cohorts and is no longer commercially available, whereas clinical evidence for the Protect PNS system is currently restricted to preliminary data, with results from ongoing prospective studies still awaited [9]. Consequently, the current evidence should be interpreted in the context of the different stages of clinical development of these systems.

Methodological heterogeneity existed across studies regarding outcome measures, stimulation protocols, follow-up duration, responder definitions, and patient reported outcome measures differed across studies, precluding direct comparison and formal meta-analysis. Moreover, most studies are prospective single-arm studies without comparison to established third-line therapies such as SNM, PTNS or intradetrusor botulinum toxin injections. The current evidence originates from a relatively limited number of investigator groups, reflecting the early stage of clinical development of these technologies. Several studies represent follow-up or secondary analyses of overlapping patient cohorts. Independent validation in future clinical studies will be important to confirm reproducibility.

Although improvements in patient-reported outcome measures, including QoL, were reported, qualitative evidence exploring patients’ experiences with these devices remains scarce. Future research should extend beyond patient related outcome measures to evaluate patient perspectives on device usability, treatment burden, home-base therapy and patient preferences, thereby providing a more comprehensive assessment of patient centered outcomes.

The investigated systems differ in their stage of clinical evaluation and regulatory development, which should be considered when interpreting the currently available evidence. The Revi system (BlueWind Medical) received CE marking in 2016 and FDA approval in 2023. The Protect PNS system is currently undergoing FDA evaluation for market approval for the treatment of OAB. The Urgent-SQ system was evaluated only in early pilot studies and did not progress toward regulatory approval.

## Conclusion

iTNM systems incorporating an anklet stimulator demonstrated promising efficacy and favorable long-term safety outcomes in patients with rOAB and UUI. Across the currently investigated systems, clinically meaningful and durable improvements were observed in urinary symptoms and QoL, while AE were predominantly mild and transient. The ability to provide minimally invasive, wireless, home-based, and patient-tailored therapy may offer important advantages over conventional PTNS and other neuromodulation strategies by reducing long-term treatment burden. Despite limitations related to heterogeneity in study design, stimulation protocols, outcome reporting, and relatively small study populations, the available evidence supports iTNM as a valuable addition to the third-line treatment landscape for OAB and UUI patients.

## Data Availability

The data supporting the findings of this study are available within the article and its referenced sources. No new datasets were generated.

## Abbreviations

AE: Adverse event
BMI: Body mass index
FU: Follow-up
HRQL: Health-related quality of life
ICIQ-FLUTS: International Consultation on Incontinence Modular Questionnaire – Female Lower Urinary Tract Symptoms
I-QoL: Incontinence Quality of Life questionnaire
iTNM: Implantable tibial neuromodulation
LUTD: Lower urinary tract dysfunction
LUTS: Lower urinary tract symptoms
MID: Minimal important difference
mo: Months
N.R.: Not reported
OAB: Overactive bladder
OAB-q: Overactive Bladder Questionnaire
pIPG: Percutaneous implantable pulse generator
PGI-I: Patient Global Impression of Improvement
PPBC: Patient Perception of Bladder Condition
PPIUS: Patient Perception of Intensity of Urgency Scale
PTNS: Percutaneous tibial nerve stimulation
QoL: Quality of life
RF: Radiofrequency
rOAB: Refractory overactive bladder
SAE: Serious adverse event
SF-36: 36-Item Short Form Health Survey
SNM: Sacral neuromodulation
UDI: Urogenital Distress Inventory
UF: Urinary frequency
UI: Urinary incontinence
UTI: Urinary tract infection
UUI: Urgency urinary incontinence
yr: Year(s)
